# Emerging Ergonomics Issues and Opportunities in Mining

**DOI:** 10.3390/ijerph15112449

**Published:** 2018-11-03

**Authors:** Patrick G. Dempsey, Lydia M. Kocher, Mahiyar F. Nasarwanji, Jonisha P. Pollard, Ashley E. Whitson

**Affiliations:** Pittsburgh Mining Research Division, National Institute for Occupational Safety and Health, Pittsburgh, PA 15236, USA; muo8@cdc.gov (L.M.K.); wgn8@cdc.gov (M.F.N.); jni3@cdc.gov (J.P.P.); nrn6@cdc.gov (A.E.W.)

**Keywords:** ergonomics, mining, human factors, occupational safety and health

## Abstract

Ergonomics is the scientific discipline that investigates the interactions between humans and systems to optimize both human and system performance for worker safety, health, and productivity. Ergonomics is frequently involved either in the design of emerging technologies or in strategies to alleviate unanticipated human performance problems with emerging technologies. This manuscript explores several such emerging issues and opportunities in the context of the mining sector. In mining, the equipment, tools, and procedures have changed considerably and continue to change. Body-worn technology provides a number of opportunities to advance the safety and health of miners, while teleoperation and autonomous mining equipment stand to benefit significantly from ergonomics applications in other sectors. This manuscript focuses on those issues and opportunities that can impact the safety and health of miners in the near term.

## 1. Introduction

Mining has long been characterized by strenuous work carried out in demanding environments underground and at surface facilities and pits. By their nature, these environments are continuously changing as mining advances, with significant variability within and between mines in work system features such as walkways, height and width of passageways, and environmental conditions. In addition to the natural environment, miners also interact with large and complex equipment, processing and preparation plant control systems, as well as myriad other systems with perceptual and cognitive demands that are required to operate a mine. The fit between miners’ capabilities and limitations and the demands of these work systems is a focus of ergonomics, which seeks to optimize human performance (including productivity, health, and safety).

There has been fairly extensive research on implementing ergonomics in mining (see Sanders and Peay (1988) and Simpson et al. (2009) for more comprehensive reviews [1,2]), although not as much research and practice as in sectors such as manufacturing and the military. There is also contemporary research on diverse topics such as developing ergonomics assessment tools for the industry [3], implementing participatory ergonomics programs in mining [4], and ergonomics approaches to designing mining equipment [5]. Ideally, ergonomics should be integrated at the design stage, but unfortunately this is rarely achieved (mining is not unique in this regard). Additionally, like other sectors, mining continues to see an evolution of processes and equipment that generates ergonomics challenges. Likewise, new technologies are being or could be introduced into the mining industry that have the potential to reduce or eliminate health and safety issues. There is also an opportunity to learn from past and current ergonomics research in other sectors that has direct relevance to technologies being adapted for mining applications.

### Objectives

The objective of this manuscript is to explore the potential ergonomics implications of emerging technologies in the mining industry. The implications include research to address potential negative consequences of emerging technologies on mine workers, as well as research to investigate the potential benefits of applying new technologies in the mining industry to increase miners’ safety and health. While not all potential ergonomics implications can be explored, we will focus on challenges and opportunities with near-term potential for impacting health and safety.

## 2. Teleoperation and Office Workstations

Teleoperation of mining equipment can have significant health and safety benefits by allowing the miner to operate equipment from a safe location without exposure to hazards inherent in many mining operations. Teleoperation has also been investigated as an option for mine rescue equipment to enter mines following a fire or explosion when the atmosphere is not hospitable to mine rescue teams (e.g., James et al., 2011 [6]). Rather than operating the equipment directly, operators are stationed at a remote location where they operate the machinery using remote controls and video or other feedback sent from the machine to the operator’s location.

One particular area of ergonomics research and practice that will be relevant to mining operations with teleoperators of mobile equipment or process control is the design of workplaces for the operators. As teleoperation of remote equipment and monitoring of autonomous vehicles become more prevalent, some operators will transition to workstations more akin to an office than a cab on a piece of machinery. Operator compartments on existing equipment have limited space and are difficult to modify to address ergonomics deficiencies. A significant advantage of teleoperation will be that remote workspaces can be designed according to ergonomics principles for both equipment [7] and workstation [8] design.

As concerns intensify over the possible health effects of sedentary work, there have been significant efforts to better understand how to alternate between sitting and standing (e.g., Karakolis and Callaghan, 2014 [9]), as well as designing workplaces to encourage more movement [10]. Teleoperation offers the opportunity to design and arrange equipment controls and displays according to ergonomics principles as well as design the workplaces to reduce sedentary behavior. Although there is no agreed duration for bouts of sitting and standing, since both prolonged sitting and standing have negative health effects, the ability to change postures and avoid prolonged static postures are two key principles of ergonomic workstation design. Teleoperation offers the ability, for example, to have operators switch tasks or take breaks to allow operators to move. Such administrative controls are difficult to achieve during typical equipment operation due to space restrictions. However, as with other safety and health considerations, addressing the ergonomics issues during the design phase is the most efficient and effective approach. While there may be a natural tendency to replicate the operator’s cabin for teleoperation, taking advantage of additional space can result in a more comfortable and efficient workstation. Mining can take advantage of several decades of research in these areas.

## 3. Body-Worn Technology

Collecting human performance data from miners performing their jobs has been extremely challenging for our research group. For example, efforts to measure and summarize postures and activities of maintenance and repair workers at mine sites using goniometers and in-shoe pressure sensors proved to be difficult, with externally-worn goniometers failing due to harsh conditions. This ultimately led to a video-based approach that was time consuming due to the amount of coding from video required [11]. In other cases, we utilized laboratory simulations to study mining tasks (e.g., Nasarwanji et al., 2016; Pollard et al., 2015 [12,13]) because the nature of the data acquisition equipment used was too delicate for use in mining environments. Emerging body-worn technologies have potential to extend field data collection of human performance data.

New wearable technologies that can collect human performance data, monitor ambient conditions, extend human capabilities, or mitigate the negative effect of activity on the body during work and leisure activities are developing at a rapid pace. These technologies have the potential to advance our ability to acquire directly-measured data for research purposes, reduce task and job demands on miners, and to provide health and safety information to mine workers. As these technologies continue to shrink in size and become more accurate, the limitations discussed in the previous paragraph may be diminished or eliminated.

### 3.1. Sensors

As technology advances, body-worn sensors are increasing in popularity. Because sensors can be worn for continuous monitoring [14], they can provide miners and occupational safety and health professionals with real-time information about environmental and physical hazards. Wearable sensors comprise three main parts: a sensing block that collects data, a communication block that transmits data, and a signal converter that transforms data into an applicable format [14]. While there are many different types of sensor blocks, sensors suitable for application in mining include environmental sensors, physiological and biochemical sensors, and motion sensors.

Environmental sensors can be used to measure temperature, humidity, and the presence of gas in a work environment [15]. Physiological and biochemical sensors can measure blood pressure, pulse rate, body temperature, and respiration rate [16,17]. Lastly, motion sensors can consist of inertial measurement units (IMUs), smart textiles, or global positioning systems (GPS). IMUs can measure gait stability and estimate fall risk [18], smart textiles can measure respiratory rate or joint angles [19,20], and GPS can be used to identify the location of a worker and warn of hazardous environments [21]. While a survey of occupational safety and health professionals indicated growing interest in incorporating wearables in the workplace, this technology has not yet been completely adopted by industry [22], nor have the potential challenges been realized that will need to be addressed as interest continues to grow.

Wearable sensors, like other new technologies, are continually developing and are not without limitations. For example, postural data and joint kinematics have historically been very difficult to collect in field conditions. Although technologies such as IMUs are less expensive, more portable, and easy to set up, they are also prone to interference from magnetic fields [23]. Inertial sensors are also prone to drift [24]. These can be significant limitations depending on where the sensors are used and the type of data required. However, the reliability and validity of these data acquisition systems continue to improve, which will increase their utility for research purposes.

The introduction of new wearable sensors in the workplace can introduce problems with data fusion and algorithms, mining suitability, and device perception in the mining community. While data fusion can provide a better representation of a worker’s activity or environment, in some situations multiple devices may be worn and methods to evaluate multi-sensor data need to be considered [25,26]. Similarly, complex algorithms can be used to estimate risks in industry, such as worker whole-body fatigue [27], heat stress [28], fall risk [18], and real-time assessment of risky postures [29]. Future research will need to test the accuracy of the algorithms, combine multi-sensor data to evaluate mining tasks, and develop sensors that can collect the required data in a comfortable and durable unit fit for the mining industry.

Until recently, most of the research for wearable sensors was home-based or from laboratory tests; more research is needed to assess the durability and accuracy of these devices in a mine setting. Wearable sensors can be constructed in many different ways including screen printing, droplet-based printing, lithography printing, and the pick-and-place method on many different substrates or textiles [17]. It is unknown whether sensor constructions will hold up in harsh work environments while still providing accurate data. Besides environmental stress, wearable sensors need to be tested for accuracy during different levels of work load and intensity [30]. There will also be permissibility requirements if sensors are used near the face of underground coal mines.

Lastly, perception of wearable sensors may be an issue with the adoption of this new technology in mining. Recent studies have examined occupational safety and health professionals and construction worker’s perception of wearable devices. Employees’ willingness to adopt wearables, specifically a GPS smart vest and smart wrist band, were affected by their perceived usefulness of the sensor, social influence, and perceived privacy risk [21]. While concerns varied for occupational safety and health professionals based on type of industry, privacy was cited as their primary concern when implementing wearable sensors for employees [22]. Perception and privacy, among the other emerging issues discussed, will need to be evaluated as additional wearable sensors are introduced and adopted by the mining industry. Privacy and the perception of privacy may need to be additional aspects of design to be addressed separately or in addition to technical and wearability aspects. Similar concerns have also been identified for sensors such as those that transmit driving behavior from commercial vehicles and prior research may assist with predicting at least some concerns.

### 3.2. Exoskeletons

An emerging technology with the potential to revolutionize mining manual handling tasks is wearable exoskeletons. De Looze and colleagues (2016) define an exoskeleton as “… a wearable, external mechanical structure that enhances the power of a person [31].” As such, exoskeletons are designed to augment and supplement humans by reducing biomechanical loads, thereby reducing risks for musculoskeletal disorders (MSDs) [32]. There are two types of exoskeletons: active and passive. Active exoskeletons use actuators to augment human power. Passive exoskeletons use materials and springs/elastic members to store and release energy during movement, and are currently being used in many applications including rehabilitation [32]. Active exoskeletons are still undergoing research and development even while in use in the aircraft and automotive manufacturing industries [33].

While active exoskeletons have been shown to significantly reduce the biomechanical loading associated with a task, there are some known issues with their application. Some designs have been found to increase tension in the back extensors while providing upper body support, increase lower body stress while providing back support, and to result in contact pressure discomfort to the wearer due the design of the attachments [34]. Weston and colleagues (2018) examined torso muscle forces and spinal loads of participants utilizing an exoskeletal vest with a mechanical arm to operate a tool [35]. They found that muscle forces in the torso extensors and the compressive spinal loads increased while wearing the exoskeleton. Without allowing for proper rest and recovery from the use of this exoskeleton, it could put the lower back at risk while redistributing the load from the arm [35].

An important factor when considering whether exoskeletons are accepted and used by workers is the level of discomfort, which could be indicated by contact pressure. Researchers have examined an active exoskeleton during lifting movements and measured contact pressure along with musculoskeletal activity [32]. The researchers determined that the exoskeleton reduced the musculoskeletal activity of the trunk as designed, and did not add any perceived effort to the legs. Furthermore, it was found that the device did not have high-contact pressure levels, but was not tested for extended durations [32]. This study suggests that it may be important to investigate why certain exoskeletons are successful at not introducing discomfort to more rapidly understand the characteristics responsible for positive and negative responses.

Research available on active exoskeletons involves short-term applications in controlled settings. The long-term risks and benefits of exoskeleton usage are not known. The effects of this mechanical assistance on the overall fitness, strength, and production capability of the user over time may result in a worker developing greater future risk for injury due to weakness. With less force required for a task, the task may become more frequent, resulting in greater risk for overuse injuries. The design and adjustability of an exoskeleton may limit the potential users, resulting in worker selection issues that may not be amenable to job-rotation strategies routinely employed for high-stress activities in mining. Space and clearance requirements for exoskeletons may require unusual postures or motions for the user. Moreover, the overall safety of the exoskeleton in an emergency situation or during the failure of any component will need to be evaluated for the mining environment.

While the designs of exoskeletons are showing much promise, the usability and organizational aspects require more research, with much of the research and development focused on the technical requirements of developing a working technology [36]. Research will be needed to ensure the proper exoskeleton is selected for the task and that the exoskeleton does not create new risks for the user. Active exoskeletons are continuing to evolve for specific work activities and this emerging technology provides significant opportunities to improve mine worker safety.

## 4. Automation and Autonomous Systems

Automation is perhaps the most complicated topic discussed thus far from an ergonomics perspective. The classic ergonomics consideration when implementing automation was the allocation of function between humans and machines based on the relative advantages of each [37]. Factors such as speed, power, and repetition favored machines, while human intelligence, fine manual manipulation, and the ability to adapt were far superior to machines. Allocation of function continues to be debated. Automation capabilities continue to increase as a result of advances in machine sensing and vision that can tilt the advantages between human and machines in one direction. Hardware and software advances have greatly expanded the opportunities for introducing automation to many human-machine systems, resulting in much more complicated decisions about choosing levels of automation for different classes of functions [38].

Automation has long been known to introduce ironies as well, with Bainbridge’s (1983) classic paper outlining how automation can actually increase problems for the human operator rather than eliminate or decrease them [39]. Industrial robots are an example; for instance, a study by Karwowski et al. (1988) showed that automation in a manufacturing facility reduced materials handling injuries but increased certain types of injuries for maintenance personnel [40]. Thus, automation has to be carefully planned and unintended consequences may need to be monitored and addressed. One of the safety features added to early industrial robots was some form of automatic shutoff (e.g., light curtains, pressure-sensitive mats) that was triggered by personnel entering the robot’s space. These were developed in response to injuries and fatalities caused by inadvertent activation, sometimes as a result of actions by maintenance and repair personnel to restore a robot to working order following some form of malfunction such as a jam.

Automation has been sought as a means of improving productivity and moving the mine worker away from potential harmful conditions. Automation in the mining industry is not novel. Most mineral-processing plants and coal preparation plants, which are part of mining operations, are often partially automated, and they crush, sort, process, and blend minerals with minimal operator oversight. Like other industries, large flat-screen displays and programmable logic controllers have eliminated the need for arrays of analog and digital displays and associated individual controls. Most recent efforts have focused on the use of robotics and automation to assist with the extraction and transport of the minerals and ore. Automation is being utilized in nearly all phases of mining including extraction using automated longwalls in underground coal [41,42,43], the loading of materials using automated draglines and shovels [43], and the transport of materials in mines using autonomous haul and dump trucks [43,44,45,46,47].

The production benefits of autonomous haul trucks for haulage of minerals and ore at surface mines have also been investigated [47,48]. Recently there has been a plethora of magazine articles highlighting the use of autonomous haulage equipment at surface mines [49,50,51]. In addition, manufacturers of large haulage equipment, including Komatsu, Caterpillar, and Volvo are in the process of developing or have some level of vehicle autonomy available for haulage equipment. With advances in technology, processing power, and the availability of automation technologies, it is no surprise that equipment manufacturers and mining companies with adequate resources are investigating and implementing automation. Automated haulage equipment does offer some significant health and safety benefits along with improvements in efficiency and production. Whole body vibration in haulage equipment has been a concern for equipment operators [52,53,54], in addition to struck-by incidents due to jolting and jarring [55]. In addition, there are a large number of fatal incidents associated with haulage equipment [56]. Using automated haulage equipment eliminates the exposure of operators to some of these hazards.

While advances in automation can yield increased productivity and move workers away from harmful conditions, it raises other issues. The risks associated with maintenance work in the mining industry have been investigated [57] and the injury statistics are not surprising; production processes that have been automated will still have equipment that requires scheduled and unscheduled maintenance and repair. Partial automation of production processes has been investigated in other settings, and findings often indicate a change in risk factors of MSDs as compared to a complete elimination of the risk factors due to automation [40,58,59,60]. Although a detailed analysis of the advantages and disadvantages of automation in the mining industry has not been conducted, trends may be similar to those in other industries. The movement of material along conveyers and automated bagging and palletizing stations is available; however, material-handling injuries are still prevalent. Finally, automation may only transfer the risk to a different point in the life cycle [61], either upstream to the manufacturing and installation of the automation or downstream to the maintenance and upkeep of the automation.

Automating systems just because automation is available, or automating easy tasks leaving the more complex tasks for humans can lead to less-than-optimal automation, and is a result of poor function allocation [47,62]. As with the design and implementation of any automated systems, common issues such as reliability, trust, loss of situation awareness, and mental workload need to be considered. Addressing all these factors simultaneously requires a human–systems integration approach and the use of a human-centered design process for system development [47,63]. One essential aspect of this process is considering the entire equipment life-cycle from concept development through manufacture and maintenance, to disposal [63]. Maintenance and repair play a critical role in mining as the usable life of equipment is often long, and due to the harsh nature of the mining environment, failure of equipment is inevitable. The types of anticipated maintenance work and the musculoskeletal risks associated with them should be considered. In addition, the need for a human operator to take over should be considered, especially as skill loss could occur over a prolonged period. The skill loss is not only attributed to knowledge of the task, but learned efficiencies in performing physically demanding jobs, such as that seen in palletizing operations where the momentum of the conveyer is used to assist loading pallets at bagging operations [64]. Hence, adopting a macroergonomic or sociotechnical perspective of automated systems design and function allocation would not only improve the overall efficiency of the system, but improve the mine worker’s overall quality of life [65].

## 5. Discussion 

The stated objective of this paper was to explore the possible contributions of emerging technologies to increase the safety and health of miners within an ergonomics framework. Compared to other industry sectors such as manufacturing, there has been relatively less attention to and implementation of ergonomics in mining. However, this can be an advantage as the extensive research on basic principles and implementation in other sectors can serve as a basis for guiding research in mining. There is considerable literature on the ergonomics of automation, and many generalizable principles that apply to mining.

Table 1 gives a summary of the research and implementation topics discussed in this paper that are amenable to near-term evaluation of potential feasibility and health and safety benefits. While this list is by no means exhaustive, the table provides a summary of applications that can be investigated.

The use of sensors and exoskeletons represents an area where research is warranted to determine the viability of these technologies for enhancing miner safety and health. Exoskeletons, in particular, are being utilized more frequently in manufacturing, and the feedback from users is being used to develop exoskeletons that are more acceptable to operators. In the case of mining, specific applications will need to be investigated that are suited to exoskeleton use, such as determining if the repetitive performance of specific tasks or movements is amenable to available technology. The viability of exoskeletons in mining environments will need to be evaluated in terms of reliability and durability since mining may be more demanding on the exoskeletons than manufacturing environments and tasks. It is likely that physical augmentation and body-worn equipment will continue to evolve, thus this area of research may be relevant for the foreseeable future.

The use of body-worn sensors may have particular appeal for research purposes, although the privacy concerns of research participants will need to be considered. Collecting human performance data on miners is difficult due to the environment, and observational methods or analyses based on video have been common. The advancement and miniaturization of sensors capable of measuring kinematics and kinetics offer the potential to increase the amount of quantitative data that can be collected for biomechanical analyses, and physiologic sensors may be able to provide quantitative insights into energy demands and consumption. Slips and falls research in mining has largely been conducted in laboratories due to the sophisticated and fragile equipment required such as force plates and motion-capture systems. Using sensors to capture slips with or without falls has potential as an exposure assessment method that is economically and practically feasible.

Perhaps the biggest contribution of ergonomics to emerging technologies is that of autonomous systems and automation. Although a relatively brief overview of ergonomics issues of automation was provided, there is extensive literature both on the design of autonomous systems as well as the ergonomics issues and unintended consequences that can be created by these systems. Mining is no different from other sectors in that automation is often largely driven by the desire to increase productivity and increase the safety of the human operator by removing the individual from a hazardous environment. Mining can take advantage of past lessons learned in other sectors. Automation in mining will still require mining-specific research, but the research can focus on applied issues such as refinement and monitoring of the automated systems rather than more fundamental basic research.

### Barriers and Limitations to Implementation

As with any industry, introducing change and technology to the mining industry will require research beyond just the technical aspects of implementation from a purely ergonomics perspective. Just as implementing changes to reduce the physical demands of mining tasks is better accomplished with miner participation [4], the same will be true of advanced technologies. Both equipment manufacturers and mine operators will be involved in some of these changes such as automation. Implementation will also benefit from behavioral sciences research to fully understand the perceived benefits and barriers to implementation.

Another limitation is that the technologies discussed were generally not discussed in reference to a particular task or job, and implementation will require specific research with varying generalizability. For example, the authors are pursuing a feasibility study of exoskeletons for application to specific mining tasks. This will require finding one or a few mining tasks that can benefit from specific exoskeletons, and that the exoskeleton will be feasible for the environment in which the task is (or tasks are) performed. A formal study will then need to be conducted to measure the acute physiological and biomechanical responses of miners to performing the task(s) with and without the exoskeletons. More broadly, automation will also need to be researched and not all mines will be appropriate based on size or other constraints. In all cases, the authors feel that the earlier in the process that ergonomics is considered, the greater the likelihood of success. Emerging technologies almost always present opportunities as well as challenges, but the authors feel that the technologies outlined in this manuscript are worth pursuing to increase the safety and health of miners.

## 6. Conclusions

A benefit of the technologies discussed in this paper is that they have the potential to increase the percentage of the population accommodated by the respective mining tasks, which is a fundamental goal of ergonomics. This is important as the miner population becomes more diverse from the standpoints of age, gender, and anthropometry. For example, equipment designed in the U.S. may be tailored to the anthropometry of the U.S. mining population. This may result in mismatches for miners in other countries. However, teleoperation affords the opportunity to design adjustable workstations appropriate for diverse populations. Similarly, exoskeletons may help reduce demands on miners also increasing the percentage of a particular miner population that can safely and comfortably perform a task or job. Overall, increased attention to ergonomics will better enable mines across different geographic regions to respond to changing production demands and workforce characteristics.

## Figures and Tables

**Table 1:** Mining scenarios amenable to ergonomics research and practice.

Research/Implementation Topic	Mining Scenarios
Workstation design	a. Equipment teleoperation workstations b. Plant control rooms c. Crusher operator compartments
Body-worn motion sensors	a. Trip and fall exposure assessment for ambulatory miners b. Continuous recording of joint kinematics and postures while performing mining tasks
Body-worn physiological and chemical sensors	a. Metabolic demands of repetitive tasks (e.g., shoveling, palletizing) b. Thermal responses to working in hot mining environments c. Sensors to monitor ambient environment for threats to safety and health
Passive exoskeletons	a. Jobs requiring repetitive motions and forceful exertions of particular joints such as the shoulder (e.g., hanging cable underground) or back (e.g., palletizing, shoveling)
Automation and autonomous systems	a. Monitoring and control of autonomous haul and dump trucks b. Control rooms c. Underground longwall systems d. Automated materials handling and palletizing equipment
